# Synergistic effect of methyljasmonate and cyclodextrin on stilbene biosynthesis pathway gene expression and resveratrol production in Monastrell grapevine cell cultures

**DOI:** 10.1186/1756-0500-1-132

**Published:** 2008-12-22

**Authors:** Diego Lijavetzky, Lorena Almagro, Sarai Belchi-Navarro, José M Martínez-Zapater, Roque Bru, Maria A Pedreño

**Affiliations:** 1 Departamento de Genética Molecular de Plantas, Centro Nacional de Biotecnología, Consejo Superior de Investigaciones Científicas (CSIC), C/Darwin 3, 28049 Madrid, Spain; 2 Cátedra de Química Orgánica y Biológica, Facultad de Ciencias Agrarias, Universidad Nacional de Cuyo-Consejo Nacional de Investigaciones Científicas y Técnicas (CONICET), M5528AHB Chacras de Coria, Argentina; 3 Departamento de Biología Vegetal, Facultad de Biología, Universidad de Murcia, Campus de Espinardo, E-30100 Murcia, Spain; 4 Instituto de Ciencias de la Vid y el Vino (Consejo Superior de Investigaciones Científicas, Universidad de La Rioja, Gobierno de La Rioja), Campus de la Universidad de La Rioja, C/Madre de Dios 51, 26006 Logroño, Spain; 5 Departamento de Agroquímica y Bioquímica, Facultad de Ciencias, Universidad de Alicante, Apartado 99, E-03080 Alicante, Spain

## Abstract

**Background:**

Plant cell cultures have been shown as feasible systems for the production of secondary metabolites, being the elicitation with biotic or abiotic stimuli the most efficient strategy to increase the production of those metabolites. Vitaceae phytoalexins constitute a group of molecules belonging to the stilbene family which are derivatives of the *trans*-resveratrol structure and are produced by plants and cell cultures as a response to biotic and abiotic stresses. The potential benefits of resveratrol on human health have made it one of the most thoroughly studied phytochemical molecules. The aim of this study was to evaluate the elicitor effect of both cyclodextrin (CD) and methyljasmonate (MeJA) on grapevine cell cultures by carrying out a quantitative analysis of their role on resveratrol production and on the expression of stilbene biosynthetic genes in *Vitis vinifera *cv Monastrell albino cell suspension cultures.

**Findings:**

MeJA and CD significantly but transiently induced the expression of stilbene biosynthetic genes when independently used to treat grapevine cells. This expression correlated with resveratrol production in CD-treated cells but not in MeJA-treated cells, which growth was drastically affected. In the combined treatment of CD and MeJA cell growth was similarly affected, however resveratrol production was almost one order of magnitude higher, in correlation with maximum expression values for stilbene biosynthetic genes.

**Conclusion:**

The effect of MeJA on cell division combined with a true and strong elicitor like CD could be responsible for the observed synergistic effect of both compounds on resveratrol production and on the expression of genes in the stilbene pathway.

## Background

The more relevant Vitaceae phytoalexins comprise a group of molecules belonging to the stilbene family [1,2], which are derivatives of the *trans*-resveratrol structure (3,5,4'-trihydroxystilbene). In addition to *trans*-resveratrol derived molecules, other oligomers produced by its oxidation and generically known as viniferins have been found as the result of infection or stress [3]. Different naturally occurring stilbenes like resveratrol, pterostilbene, piceatannol and resveratrol glucoside derivatives [4] are known to be strong antioxidants. In particular, the potential benefits of resveratrol on human health have made it one of the most thoroughly studied phytochemical molecules [5]. See de la Lastra and Villegas [6] for a review of the reported resveratrol effects.

Stilbenes are synthesized via the phenylpropanoid/malonate pathway from phenylalanine that, in turn, is converted into cinnamic acid by phenylalanine ammonia lyase (PAL). The consecutive action of cinnamate 4-hydroxylase (C4H) and 4-coumarate CoA ligase (4CL) transform cinnamic acid into *p*-coumaryl-CoA. Derived compounds, collectively referred to as polyphenols, are originated at this branching point through the action of enzymes chalcone synthase (CHS) and stilbene synthase (STS) for flavonoids and stilbenoids, respectively [2].

*Vitis vinifera *cell cultures have been used in several studies to explore the factors involved in the induction and regulation of stilbene biosynthesis and metabolism [7,8]. Jasmonic acid (JA) and its more active derivative methyljasmonate (MeJA) have been proposed as key compounds of the signal transduction pathway involved in the elicitation of secondary metabolite biosynthesis which takes part in plant defence reactions [9]. Application of MeJA on grapevine leaves and plant cell suspension cultures can induce the accumulation of stilbenes [10,11]. However, the reported amount of stilbenes secreted to the medium in MeJA-treated cell cultures is negligible [8,12].

Cyclodextrins (CDs) are naturally occurring cyclic oligosaccharides derived from starch. Addition of 2,6 dimethyl-β-ciclodextrin (DIMEB) to grapevine cell cultures induces both resveratrol biosynthesis and its accumulation in the culture media [13]. Among the differently modified β-cyclodextrins, the methylated and hydroxypropylated caused the highest production of this phytoalexin, which is translocated to the cell walls and accumulates in the culture media [14,15].

The aim of this study was to evaluate the elicitor effect of joint applications of CDs and MeJA on grapevine cell cultures by carrying out a quantitative analysis of their role on resveratrol production. We also monitored the expression of several genes encoding key enzymes in the phenylpropanoid pathway, including those involved in resveratrol biosynthesis, to determine the relationship between resveratrol accumulation in the medium after elicitation and the regulation of gene expression. A synergistic interaction between CD and MeJA on resveratrol production and on the expression of stilbene biosynthesis related genes is discussed.

## Results

### MeJA but not CD affects cell growth

**Figure 1:**
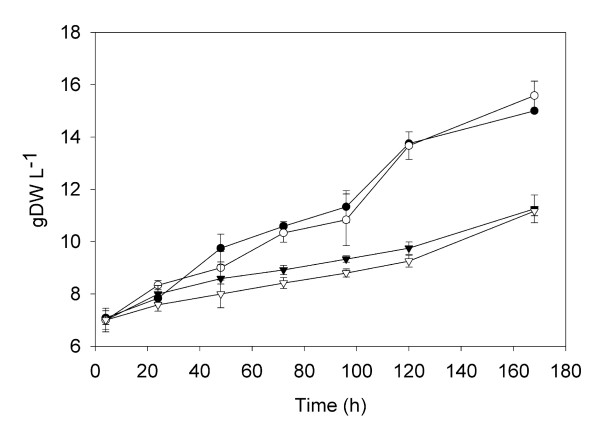
**Growth curves of grapevine treated cell suspension cultures**. (solid circle) Control cells, (open circle) CD treated cells, (solid triangle) MeJA treated cells, (open triangle) CD+MeJA treated cells. Measurements are expressed as g DW l^-1 ^and values are given as the mean ± standard deviation of three replicates.

Grapevine cell cultures were treated with DIMEB (CD), methyljasmonate (MeJA) and a combination of CD and MeJA. As shown in Figure 1, CD-treated cells displayed a similar sustained biomass increase (from 7 to over 15 g DW l^-1^) and growth curve as control untreated cells indicating that CD treatment did not affect cell growth. On the other hand, cell cultures treated with MeJA alone or together with CD showed a growth curve (Figure 1) with significantly lower biomass generation (up to 30% less than control and CD-treated cells). This biomass reduction did not result from massive cell lyses, since no losses in cell viability could be observed by fluorescent microscopy (Additional File 1).

### CD and MeJA synergistically induce resveratrol production

**Figure 2:**
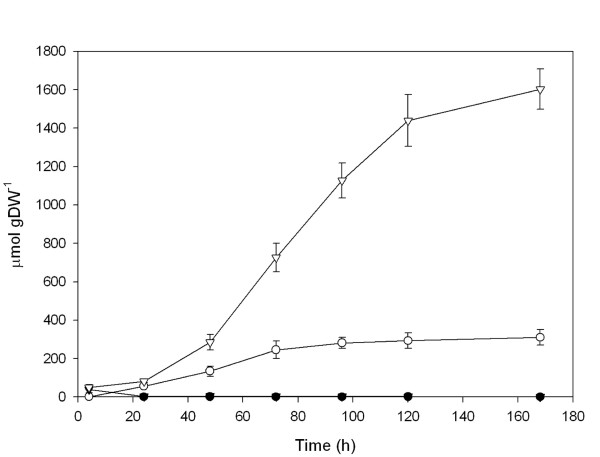
**Resveratrol accumulation of grapevine treated cell suspension cultures**. (solid circle) Control cells, (open circle) CD treated cells, (solid triangle) MeJA treated cells, (open triangle) CD+MeJA treated cells. The accumulation of *trans*-resveratrol in the spent medium was measured as μmol g DW^-1 ^and values are given as the mean ± standard deviation of three replicates.

To compare the effects of the different treatments in *trans*-resveratrol production, the level of this compound was analyzed in the spent medium at different times. Trace amounts of *cis*-resveratrol were also detected (Additional File 2). As shown in Figure 2, no significant amounts of *trans*-resveratrol were detected in the spent medium when cell cultures were elicited with MeJA. In contrast, CD treated cell cultures showed a significant production of resveratrol. The level of resveratrol increased linearly until 72 h, and then remained constant for the rest of the experiment until 168 h (Figure 2). Remarkably, when cells were simultaneously elicited with CD and MeJA, the accumulation of resveratrol in the medium increased exponentially reaching a concentration plateau after 120 h and a final concentration higher than 1600 μmole gDW^-1^, which almost represents one order of magnitude higher than the final concentration obtained in CD treated cells (Figure 2 and Additional File 2). The content of stilbenoids within cells (free and glycosilated forms of *trans*- and *cis*-resveratrol) was also analyzed (Additional File 3). No statistically significant differences were found in the total amount of intracellular stilbenoids between MeJA treated cells and the control, while the CD and CD + MeJA treated cells presented a 3- and 20-fold increment in relation to the control cells, respectively. The *trans*-isomers represented between 80 to 90% of intracellular stilbenoids. However, as compared to the overall production, endogenous stilbenoids represent less than 1% of the total stilbenoids. Therefore, the extracellular *trans*-resveratrol correlates with the actual biosynthetic activity of the cells.

### CD and MeJA synergistically and specifically induce the expression of STS and the general phenylpropanoid pathway

**Figure 3:**
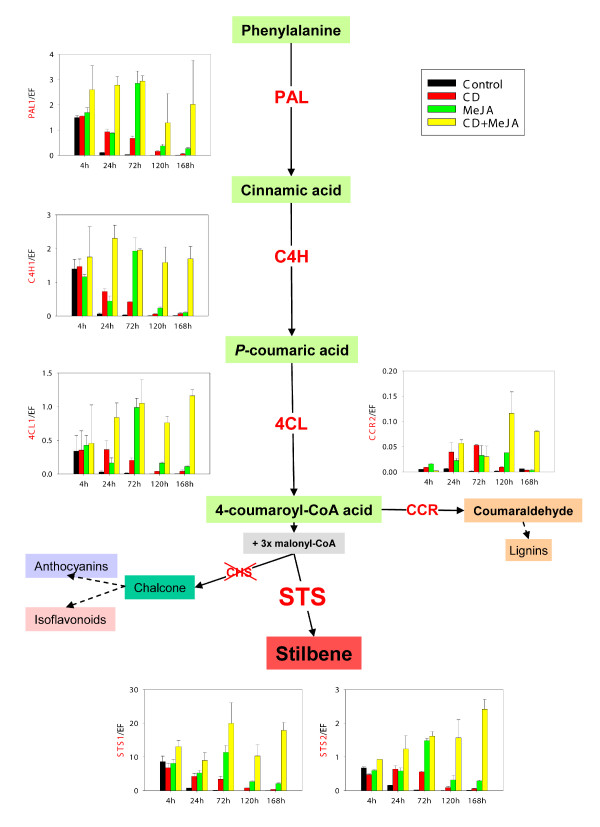
**Relative expression of phenylpropanoid-related genes in grapevine treated cell suspension cultures**. PAL, C4H, 4CL, STS, CHS and CCR transcribed mRNAs were analysed by real-time quantitative RT-PCR. Levels of transcripts were calculated using the standard curve method with grapevine EFα1 gene as internal control. Values are given as the mean ± standard deviation of three replicates.

In order to analyze the relationship between levels of phytoalexin accumulation in the medium and the expression of related biosynthetic genes, we performed real time qRT-PCR analyses of two stilbene synthase (*STS*) genes and different genes from the phenylpropanoid pathway. The relative expression levels of these two *STS *genes were quantified at five different time points through the incubation of the cell cultures with CD, MeJA or the combined treatment with CD and MeJA. Both *STS *genes showed the same expression profile in all the treatments, although *STS1 *expression was one order of magnitude higher than *STS2 *(Figure 3). In control cell cultures and in all the treatments, *STS *expression was higher at 4 h, presumably as a consequence of the treatments setup. In agreement with this possibility, *STS *expression in control cells dropped 24 h after the treatment and almost disappeared during the rest of the incubation. In contrast, 24 h after the treatment, we detected a marked expression of *STS *in CD treated cells, slowly decreasing until the end of the experiment (Figure 3). Expression of *STS *in the MeJA incubated cells at 24 h was similar to that observed in the CD treatment but rose significantly at 72 h to reach its maximum values. The more notable results were observed in cell cultures elicited with both CD and MeJA. Expression of *STS *was higher in this treatment than in all the other treatments at all time points (Figure 3). Similarly to what was observed in the MeJA treatment, a maximum of expression of both *STS *genes was observed at 72 h. Later expression stabilized (or even decreased) at 120 h to increase again at the last analyzed point 168 h. During the first three days of treatment, expression of *STS *in the combined treatment was relatively similar to the sum of the CD and MeJA effects. However, after 120 h, the combined treatment of CD and MeJA yielded expression values that seem to reveal a synergistic interaction between those two compounds (Figure 3).

To evaluate the specificity of the stilbene pathway induction, we analyzed the relative expression of the general phenylpropanoid pathway genes (*PAL*, *C4H*, *4CL*) as well as that of *CCR *and *CHS *in treated cell cultures. These last two genes encode key enzymes of stilbene alternative pathways: *CHS *catalyzes the first step in the anthocyanin and isoflavonoid biosynthetic pathways, and *CCR *plays a similar role in the lignin biosynthetic pathway. Gene expression was evaluated at the same time points described above. The expression profiles of *PAL*, *C4H*, and *4CL *were almost identical to those of *STS *with an earlier induction in the case of the combined treatment of CD and MeJA (Figure 3). On the other hand, the relative expression of *CCR *was very low, about one order of magnitude lower than that of the less expressed gene analyzed in the pathway (i.e. *4CL*, Figure 3). Moreover, none of two CHS assayed genes displayed any detectable expression along the whole experiment (Figure 3).

## Discussion

The results described here show that the combined addition of MeJA and CD to *V. vinifera *cv. Monastrell albino cell cultures yields a much higher resveratrol accumulation than the sum of the individual additions. In our experiments, the combined treatment increased seven times the yield of resveratrol when compared to CDs alone. This final resveratrol level (1600 μmole gDW^-1^) represents an increase between 10- and more than 1000-fold with respect to previous reports [8,12,16]. The expression analysis of this response shows that both elicitors stimulated the expression of *PAL*, *C4H*, *4CL *and *STS *independently of the anthocyanins/isoflavonoids (CHS) and lignins (CCR) pathways and therefore both induce stilbene biosynthetic genes in a highly specific way, in agreement to results reported by Saigne-Soulard et al.[17]. Furthermore, the synergistic interaction of both elicitors on resveratrol production (Figure 2) seems to be the result of their synergistic effect on the expression of biosynthetic genes (Figure 3).

In MeJA-treated cells, a significant reduction in cell growth was observed (Figure 1) in parallel with a strong induction of the general phenylpropanoid pathway (Figure 3), as recently reported for *Arabidopsis *cell suspension cultures [18]. However, although *STS *expression was highly induced (Figure 3), no significant amounts of resveratrol were detected in the spent media (Figure 2). Such discrepancy could be due to either post-transcriptional and/or post-translational regulatory mechanisms [12,16,19].

CD elicited cells effectively produced significant amounts of resveratrol (Figure 2) in correlation with a transient expression of the central phenylpropanoid enzymes and *STS *genes (Figure 3). Furthermore, most (or all) the resveratrol synthesized up to 72 h remained in the culture medium until the end of the assay (Figure 2). CDs are able to form inclusion complexes with stilbene compounds, such as *trans-*resveratrol and diethylstilbestrol [13,20], which could protect resveratrol from oxidation or glucosylation. This could explain the observation that, although *STS *expression dropped after 72 h in the CD treatment (Figure 3), the amount of resveratrol stayed constant (Figure 2). In contrast to the effect of MeJA, CD treated cells were not altered in their growth, displaying a similar biomass growth curve as control cells (Figure 1). Since both complexed and uncomplexed CD molecules remain in the culture medium during the whole assay, the transient gene expression and the high but limited production of resveratrol must be the result of additional regulatory mechanisms. Given the regular growth curve of the CD-treated cell cultures it is tentative to propose that engagement of cells in active division could somehow compete with further production of resveratrol after a transient elicitation response. In fact, Naill & Roberts [21] observed that most metabolite productive cells in *Taxus cuspidata *suspension cultures were in the G_0_/G_1 _phase of the cell cycle (i.e. non-cycling cells) and this stage was suggested as the most specialised for accumulation of secondary metabolites.

In agreement with the previous hypothesis, when both CD and MeJA were simultaneously added to the culture medium, they caused a significant reduction in cell growth (Figure 1) as well as a sustainable maximum expression of *STS *and central phenylpropanoid genes, even after 168 h (Figure 3), which was paralleled by a maximum resveratrol accumulation in the medium (Figure 2). We believe that the blockage in cell division and metabolic rearrangement likely caused by MeJA [18] could place the cells in a non-cycling state [21] allowing a sustained elicitation by CD.

It has been suggested that MeJA may induce a subset of secondary metabolite biosynthetic genes which could modulate expression of genes and accumulation of compounds induced by elicitors [22,23]. Although we cannot completely discard this possibility, we show that the synergistic effect observed on resveratrol production is related with a synergistic effect on the expression of the same set of stilbene biosynthetic genes induced by CD (Figure 3). The observed effects of MeJA on cell suspension growth and the recent characterization of MeJA effects on *Arabidopsis *cell cultures open the possibility to propose an alternative hypothesis to explain this synergy based on the combined effect of MeJA on cell cycle together with a true and strong elicitor like CD. Further experiments will be required to confirm this possibility on the interaction between cell cycle and secondary metabolite biosynthetic gene expression.

## Methods

### Establishment of calli and cell suspension cultures

*Vitis vinifera *L. cv. Monastrell albino calli were established as previously described [24]. Cell suspensions were established and maintained as described by Bru et al. [14].

### Elicitation of Monastrell albino cell cultures

Elicitation experiments were carried out on three replicates of 14 days old grapevine cell suspensions. Washed cells (20 g FW) were transferred into 250 ml flask and resuspended in 100 ml of sterile fresh medium containing either 50 mM DIMEB, 100 μM MeJA or 50 mM DIMEB + 100 μM MeJA. Control cultures contained no additional DIMEB or MeJA. In order to assess for any effect on cell growth of the ethanol used to deliver the MeJA, we carried out biomass measures in control cell suspensions treated with 0.2% v/v ethanol during the experimental set up. There were no significant differences in cell growth between ethanol-treated and non-treated control cell suspensions (data not shown). All cell suspensions were incubated for up to 168 h at 25°C in darkness in a rotary shaker (110 rpm) and a cell growth time course was performed both for treated and control cells. After elicitation, cells were filtered from the spent medium under gentle vacuum, rapidly washed with cold distilled water, weighted and frozen at -80°C until use. The spent medium was used for stilbenoids analysis.

### Analysis of stilbenoids in the spent medium and in cells

Samples were analyzed by liquid chromatography according to Dalluge et al. [25] with modifications in an Agilent 1100 series HPLC equipped with UV-vis and ESI-MS detectors. For more details, see Additional File 2. A time course of stilbenoid production was also performed up to 168 h of cell culture.

### RNA isolation, cDNA synthesis and Real-time quantitative RT-PCR (qRT-PCR)

Total RNA was extracted from frozen cells (0.5 g FW) by means of the TRIZOL reagent (INVITROGEN) following the manufacture's recommendations. cDNA synthesis and qRT-PCR procedures were performed according to Reid et al. [26]. Grapevine gene specific primers were designed using the Oligo Explorer 1.2 software (Gene Link). Primer sequences used in the qRT-PCR analyses are presented in Table 1. Data were analyzed using the 7300 SDS software 1.3 (Applied Biosystems). Transcript level was calculated using the standard curve method and normalized against grapevine EFα1 gene (UniGene Vvi.1750) used as reference control. Relative expression of the different genes was analyzed at five time points.

**Table 1:** Primer pairs used for real time quantitative RT-PCR

Gene abbreviation	Gene definition	GenBank accession	Unigene ID	Primer pair (5'-forward-3'/5'-reverse-3')	Product size (bp)
STS1	Stilbene synthase1	DQ366301	Vvi.8	CGAAGCAACTAGGCATGTGT/CTCCCCAATCCAATCCTTCA	134

STS2	Stilbene synthase2	DQ366302	Vvi.8	ACCAAAGTCCAAGATCACCCA/ACAACATCACCCTTCTAACCGAT	122

PAL1	Phenylalanine ammonia lyase	EC987386	Vvi.1950	CCGAACCGAATCAAGGACTG/GTTCCAGCCACTGAGACAAT	183

C4H1	Cinnamate-4-hydroxylse	EC995763	Vvi.6228	AAAGGGTGGGCAGTTCAGTT/GGGGGGTGAAAGGAAGATAT	109

4CL1	4-coumarate-CoA ligase	EC947790	Vvi.1251	CTGATGCCGCTGTTGTTTCG/GCAGGATTTTACCCGATGGA	198

CHS1	Chalcone synthase1	EC996578	Vvi.117	GTCCCAGGGTTGATTTCCAA/TCTCTTCCTTCAGACCCAGTT	157

CHS2	Chalcone synthase2	EC996527	Vvi.1973	TTTGGGCATCAAGGACTGGA/CTCGGGCTTTAGGGCTAAT	100

CCR2	Cinnamoyl-CoA reductase	CF517687	Vvi.15864	ACAGCATGACGACTCTCTTCG/AGTGACAAGGGGTGGATTGA	182

## Competing interests

The authors declare that they have no competing interests.

## Authors' contributions

DL designed the study, performed qRT-PCR and statistical analysis, coordinated the study and drafted the manuscript. LA performed the cell culture work and qRT-PCR analysis. SBN performed the cell culture work. JMMZ participated in the design of the study and the manuscript drafting. RB and MAP coordinated the cell culture work, participated in the design of the study and the manuscript drafting. All authors read and approved the final manuscript.

## Supplementary Material

Additional file 1 Cell viability of grapevine cell suspension cultures treated with MeJA. Cell viability was evaluated by incubating the cells for 1–2 min in fresh Gamborg medium containing 100 μg ml-1 fluorescein diacetate. Fluorescence was observed with a DMRB Leica microscope using a Leica filter (λexc = 490 nm, λemi = 520 nm). (a) and (b) bright field (40×), (c) and (d) UV light (40×), (e) and (f) 10× zoom-in. Click here for file

Additional file 2 Chromatographic profile of culture medium (A) and cell extract (B) at 168 h. One volume of culture medium is diluted with two volumes of water and twelve of pure methanol. Fifty mg of freeze-dried cells were extracted overnight in 4 mL methanol at 4°C. The extract was diluted with water to a final concentration of 80% (v/v) methanol. Then 30 μL of diluted medium is analyzed by HPLC. Elution of stilbenoids was recorded at 306 nm and compounds are detected by mass spectrometry. Authentic resveratrol (Sigma), *t*-Piceid (Chromadex) were used for compound confirmation and quantification. The *cis*- isomers were obtained by exposure to UV light of the *trans*- and used for quantification in the same manner. Click here for file

Additional file 3 Analysis of stilbenoids in cells at 168 h. Values (in μmole gDW^-1^) are given as the mean ± standard deviation of three replicates. For methodological details, see Additional File 2. Click here for file
