# Supplementary figures and images for: Synergistic effect of methyljasmonate and cyclodextrin on stilbene biosynthesis pathway gene expression and resveratrol production in Monastrell grapevine cell cultures
Source: BMC Res Notes. 2008 Dec 22;1:132. doi: 10.1186/1756-0500-1-132 (PMC2628674; doi:10.1186/1756-0500-1-132)

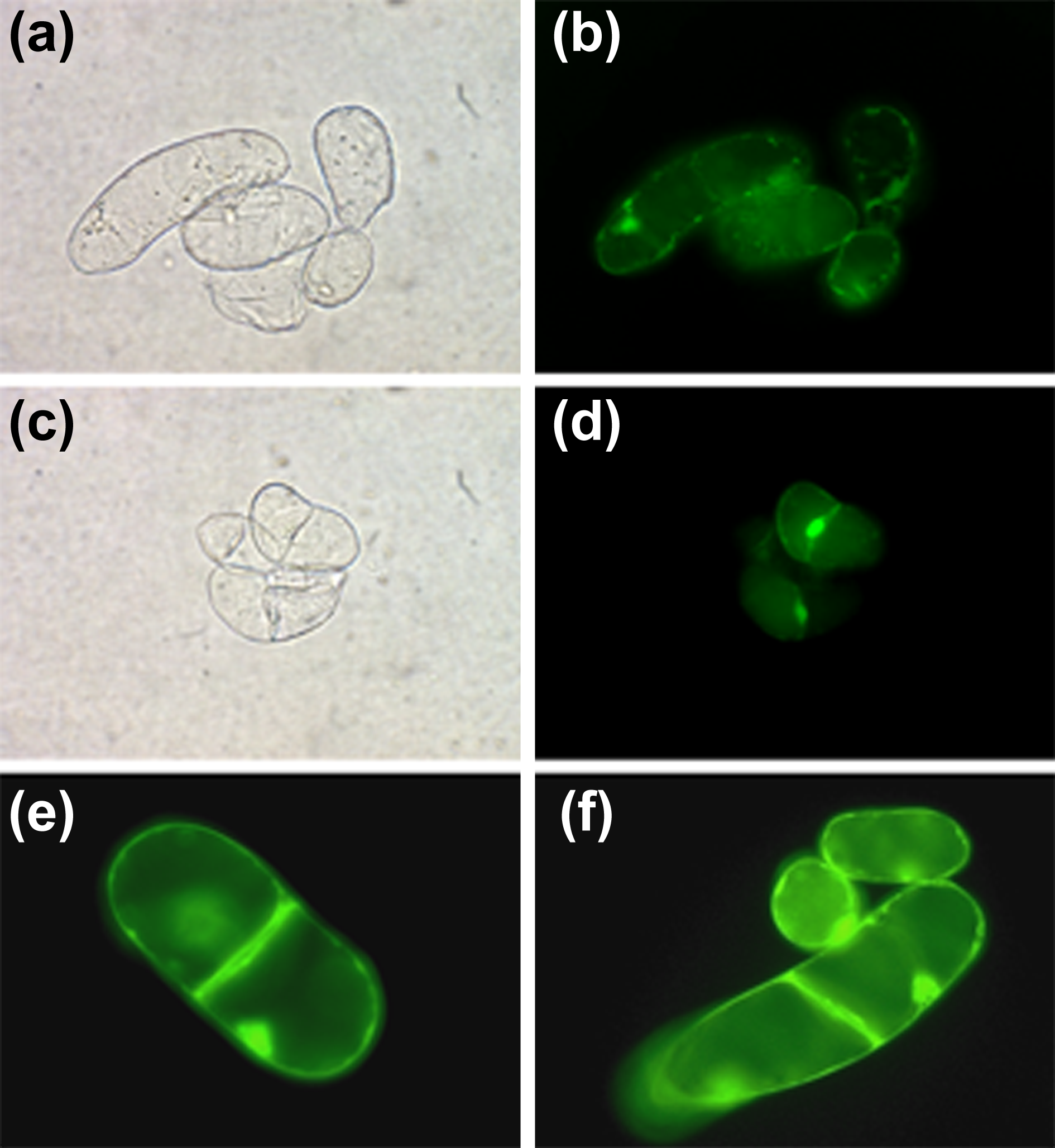

Supplement: Additional file 1 — Cell viability of grapevine cell suspension cultures treated with MeJA. Cell viability was evaluated by incubating the cells for 1–2 min in fresh Gamborg medium containing 100 μg ml-1 fluorescein diacetate. Fluorescence was observed with a DMRB Leica microscope using a Leica filter (λexc = 490 nm, λemi = 520 nm). (a) and (b) bright field (40×), (c) and (d) UV light (40×), (e) and (f) 10× zoom-in. [file 1756-0500-1-132-S1.png]

**A**

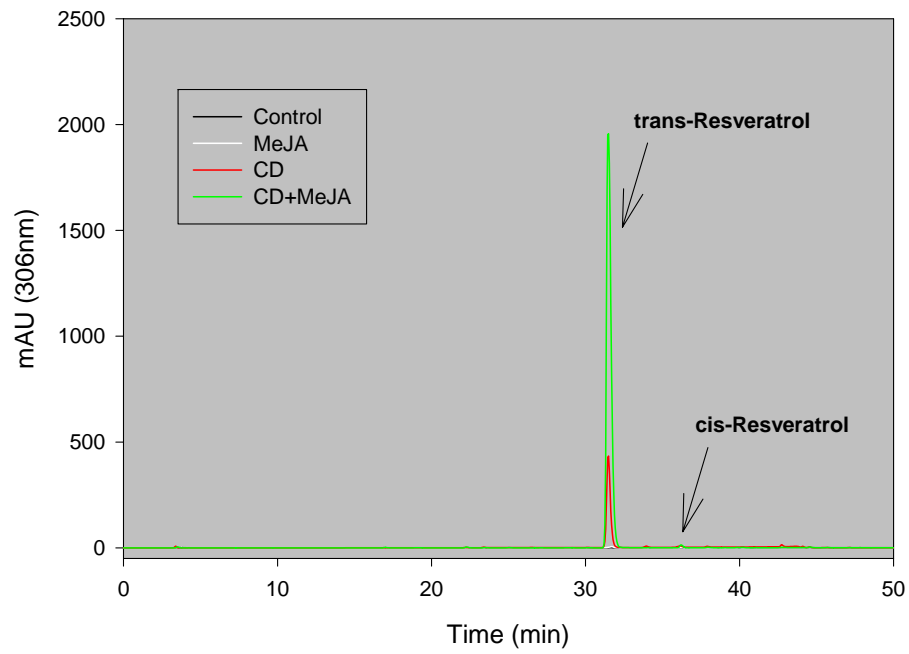

**B**

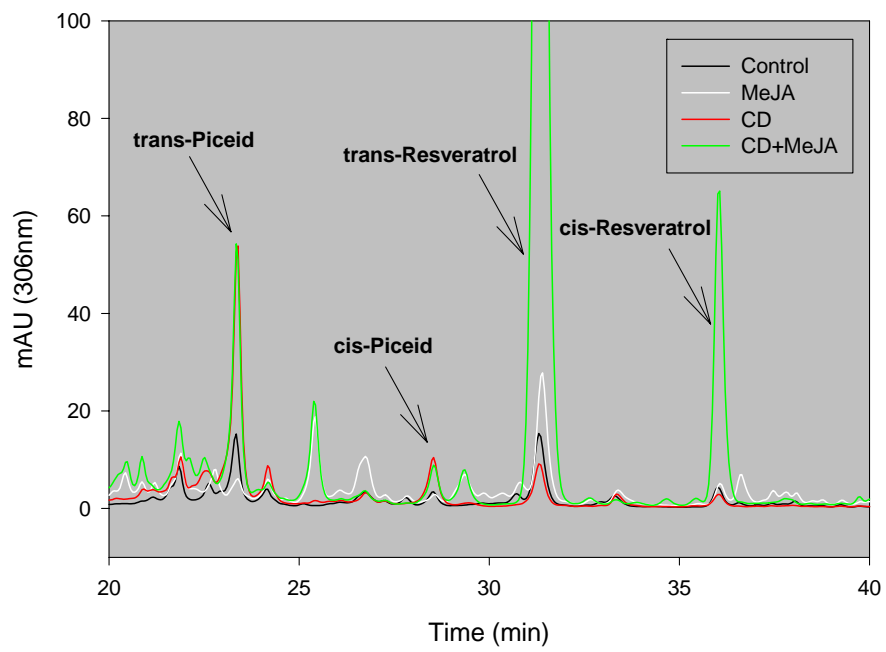

Supplement: Additional file 2 — Chromatographic profile of culture medium (A) and cell extract (B) at 168 h. One volume of culture medium is diluted with two volumes of water and twelve of pure methanol. Fifty mg of freeze-dried cells were extracted overnight in 4 mL methanol at 4°C. The extract was diluted with water to a final concentration of 80% (v/v) methanol. Then 30 μL of diluted medium is analyzed by HPLC. Elution of stilbenoids was recorded at 306 nm and compounds are detected by mass spectrometry. Authentic resveratrol (Sigma), t-Piceid (Chromadex) were used for compound confirmation and quantification. The cis- isomers were obtained by exposure to UV light of the trans- and used for quantification in the same manner. [file 1756-0500-1-132-S2.pdf]
