# Supplementary material for: Synergistic effect of methyljasmonate and cyclodextrin on stilbene biosynthesis pathway gene expression and resveratrol production in Monastrell grapevine cell cultures
Source: BMC Res Notes. 2008 Dec 22;1:132. doi: 10.1186/1756-0500-1-132 (PMC2628674; doi:10.1186/1756-0500-1-132)
Supplement: Additional file 3 — Analysis of stilbenoids in cells at 168 h. Values (in μmole gDW-1) are given as the mean ± standard deviation of three replicates. For methodological details, see Additional File 2. [file 1756-0500-1-132-S3.pdf]

	Control	MeJA	CD	CD + MeJA
trans-piceid	0.060±0.011	0.025±0.003	0.266±0.092	0.212±0.028
cis-piceid	0.017±0.003	0.014±0.003	0.072±0.012	0.041±0.009
trans-resveratrol	0.040±0.036	0.089±0.041	0.028±0.018	2.160±0.730
cis-resveratrol	0.010±0.014	0.017±0.006	0.013±0.001	0.292±0.021
Total stilbenoids	0.127±0.033	0.145±0.060	0.379±0.084	2.705±0.737
